# Antibodies targeting HSV glycoprotein B require effector functions to protect neonatal mice

**DOI:** 10.1128/jvi.00050-26

**Published:** 2026-03-09

**Authors:** Matthew D. Slein, Lesle M. Jiménez, Iara M. Backes, Evelyn M. Turnbaugh, Callaghan R. Garland, Scott W. MacDonald, Alejandro B. Balazs, David A. Leib, Margaret E. Ackerman

**Affiliations:** 1 Department of Microbiology and Immunology, Geisel School of Medicine at Dartmouth 540598, Lebanon, New Hampshire, USA; 2 Thayer School of Engineering, Dartmouth College 3728, Hanover, New Hampshire, USA; 3 Ragon Institute of Massachusetts General Hospital, Massachusetts Institute of Technology and Harvard University 1812, Cambridge, Massachusetts, USA; University of Virginia, Charlottesville, Virginia, USA

**Keywords:** antibody, herpesvirus, effector function, neutralization, mechanism, neonatal

## Abstract

Glycoprotein B (gB) serves as the viral fusion protein for herpes simplex virus (HSV), mediating fusion between viral and host membranes resulting in infection. As such, gB represents a critical target for the host immune system with high potential relevance for vaccine design. Here, we investigated the mechanisms of protection for a panel of gB-specific monoclonal antibodies (mAbs) in a mouse model of neonatal HSV (nHSV) infection. Depending on dose, viral neutralization contributed, but Fc effector functions were critical for mAb-mediated protection against nHSV mortality. Moreover, adeno-associated virus-mediated *in vivo* expression of a gB-specific mAb in mice provided transgenerational protection against HSV-1 and HSV-2 mortality. These findings demonstrate that antibodies targeting gB can serve as potent therapeutics and that they require diverse functional profiles to afford optimal protection, informing vaccine design.

**IMPORTANCE:**

Antibodies represent promising drugs for the prevention and treatment of viral infections, especially when efficacious vaccines are unavailable. Determining the dominant mechanisms of Ab-mediated protection is a critical step in the design and optimization of potential antibody therapies. In this study of antibody-mediated protection of neonatal mice from herpes simplex virus, efficacy and mechanism of action of antibodies that recognize viral glycoprotein B (gB) were dependent on dose, effector functions, and viral neutralization capacity. Overall, while viral neutralization likely contributes to monoclonal antibody-mediated protection, the ability for gB-specific antibodies to mediate Fc domain-dependent effector functions was unexpectedly crucial.

## INTRODUCTION

Unlike many other viruses, herpesviruses split entry mechanisms across several surface glycoproteins. Herpes simplex virus type 1 and type 2 (HSV-1 and HSV-2) have four glycoproteins that are required for viral entry (1): glycoproteins D, B, H, and L (gD, gB, gH, and gL). As these antigens are critical for infection, they are important targets for the host immune response. Viral entry begins with virus attachment to the cell surface by gB and gC, which contact heparan sulfate proteoglycans (2, 3). Virions then begin the fusion process after gD binds to one of its host cell receptors: nectin-1, nectin-2, HVEM, or 3-O-sulfated heparan sulfate (1). gD binding then triggers the engagement of gB and gH/gL, resulting in protein structural rearrangement between members of the fusion complex before finally leading to membrane fusion mediated by gB (4–6). Given this activity, gB has been used in past vaccine trials (7–9) with the goal of eliciting a protective neutralizing antibody response to prevent the establishment of infection.

The viral fusogen for herpesviruses, gB, is a class III fusion protein (10, 11) whose ectodomain is divided into five subdomains (11). Of these domains, DI, DII, and DIV are known targets of neutralizing Abs (12, 13), which presumably act by restricting rearrangement of the trimer between the metastable prefusion and lower-energy postfusion states to prevent viral fusion and thus viral entry (13). Neutralizing Abs may also function by blocking interactions between gH/gL and gB, which are required for the initiation of fusion with host cell membranes (5). Though considered a crucial target, mechanisms of antibody-mediated protection for gB-targeting Abs remain understudied. Generalized insights into how gB-specific monoclonal antibodies (mAbs) mediate protection against HSV infection have the potential to drive the design of improved strategies for passive and active immunization.

Abs mediate protection through both direct antiviral activities, such as neutralization mediated by blocking viral entry, and indirect antiviral activities such as antibody-dependent cellular cytotoxicity (ADCC), phagocytosis, and complement deposition, which depend on the interaction of the Fc domain with innate immune receptors. Preclinical and clinical evidence suggests that both neutralization and effector functions are important for antibody-mediated control of HSV infections (14–20). Some evidence highlighting the relative importance of effector function can be found in the protection afforded by non-neutralizing mAbs (21). A more complete dissection of the mechanisms by which gB-specific Abs, particularly those with neutralizing activity, mediate protection has yet to be performed. Defining the mechanisms of protection supports the development of mAb therapeutics that can aid in care for vulnerable populations such as neonates and immunocompromised individuals. Although relatively rare, symptomatic neonatal HSV (nHSV) infections can have devastating consequences with high mortality rates and lifelong morbidity despite small molecule antiviral therapy (22–24). The presence of maternal HSV-specific Ab dramatically reduces the risk of acquisition and severity of nHSV, supporting the notion that mAbs could be promising therapeutics for nHSV infections (25).

We previously determined that mAbs targeting the viral entry mediator, gD, require neutralization and effector functions for broad and potent protection in a mouse model of nHSV infection (14). As both gD and gB are required for viral entry, we sought to investigate whether mechanisms of Ab-mediated protection are conserved between HSV surface glycoproteins using a panel of gB-specific mAbs. By using a combination of mAbs with diverse neutralization profiles and Fc engineering, we also sought to define and generalize the mechanisms of protection for gB-specific mAbs in a mouse model of nHSV infection. We find that gB-specific mAbs can protect neonatal mice from HSV-mediated mortality through both neutralization and effector functions, with a striking requirement for effector functions at low doses of mAb. Moreover, a gB-specific mAb expressed *in vivo* by adeno-associated virus (AAV)-vectored delivery provides transgenerational protection against neonatal disease caused by HSV-1 and HSV-2.

## RESULTS

### Characterization of HSV gB-specific mAbs

**Fig 1:**
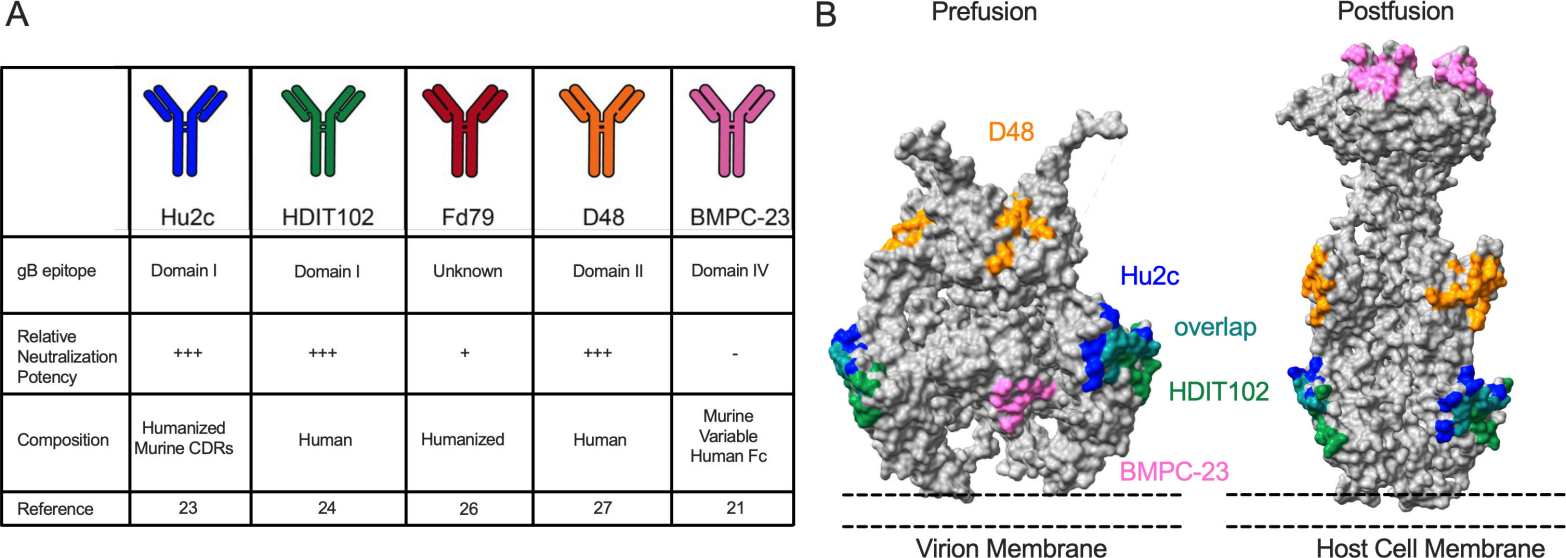
Characterization of HSV gB-specific mAbs. (**A**) Table of gB-specific mAbs used in this study. Reported binding domain on gB based on available crystal structures of mAbs. Neutralization potencies for each mAb. Origin mAb composition and original reference for each gB-specific mAb. (**B**) Epitopes for each mAb visualized on prefusion (left, PDB: 6z9m, 41) or postfusion (right, PDB: 2gum, 42) HSV-1 gB trimers. Hu2c: blue, HDIT102: green, overlap between Hu2c and HDIT102: teal, D48: orange, BMPC-23: pink.

To understand how mAbs that target gB mediate protection, we evaluated a panel of five distinct mAbs: Hu2c (26), HDIT102 (27), BMPC-23 (21), Fd79 (28), and D48 (29) (Fig. 1A). The mechanisms by which the majority of these mAbs mediate protection remain understudied and have yet to be evaluated in a neonatal model of infection. Each mAb was expressed recombinantly on a human IgG1 background. Of these, Hu2c (HDIT101) has entered clinical trials for the treatment of recurrent genital infections (NCT04165122, NCT04539483). These mAbs differ in their species of origin, relative neutralization potencies in *in vitro* assays assessing entry inhibition, and target different domains of the viral fusion protein (Fig. 1A and B). All but Fd79 have defined binding epitopes determined by structural studies (21, 27, 29). Hu2c, HDIT102, and D48 bind domains critical for viral fusion (Fig. 1A and B). Hu2c and HDIT102 both bind DI, which contains the fusion loops, and D48 binds DII, which undergoes rearrangement as a part of viral fusion (13). In contrast, BMPC-23 binds a region of DIV, which is described as inaccessible in the prefusion state (21) (Fig. 1B), consistent with its inability to neutralize HSV.

**Fig 2:**
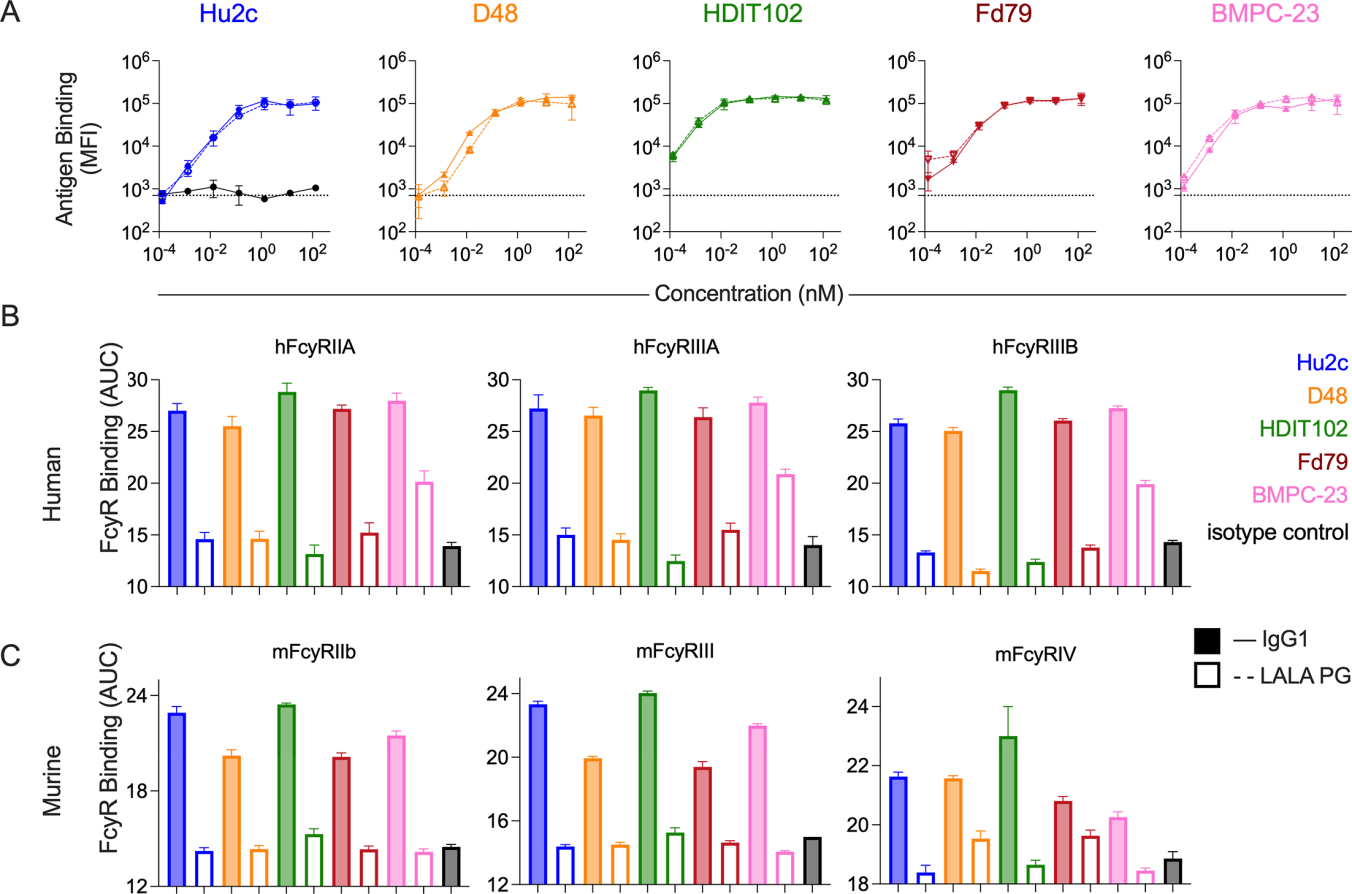
Antigen and Fc receptor binding characterization of WT and Fc-engineered gB-specific mAbs. (**A**) Median fluorescent intensity (MFI) of binding of each gB-specific mAb variant to recombinant HSV-1 gB via multiplex assay, and as compared to an isotype control (black, left panel). (**B and C**) FcγR binding profiles of each gB-specific mAb variant and isotype control (black). Bar graphs represent the area under the curve (AUC) values for binding to each of three major low to moderate affinity human (**B**) and mouse (**C**) FcγR. Error bars represent standard deviation from the mean (**A**) or standard error of the mean (**B and C**). gB binding and FcγR binding experiments were performed in technical replicate. IgG1 and LALA PG forms are depicted in solid lines with filled symbols and dotted lines with hollow symbols, respectively.

To determine the mechanisms of *in vivo* protection and dissect the relative contributions of neutralization and effector functions in mediating protection against nHSV infections in a mouse model, we engineered each gB-specific mAb to lack Fc effector functions by incorporating LALA PG (30) mutations into the Fc region. Both IgG1 and LALA PG forms of each mAb bound to recombinant HSV-1 gB (Fig. 2A), while, as expected, the isotype control HSV8, an HSV gD-specific mAb, did not. Binding to human Fcy receptors (FcyR), specifically, FcyRIIA, FcyRIIIA, and FcyRIIIB (Fig. 2B; Fig. S1A), was measured for IgG1 and LALA PG Fc function knockout versions. Whereas IgG1 forms of the gB mAbs tested bound all FcyR tested, the LALA PG versions of the gB mAbs had diminished binding. Intriguingly, BMPC-23 LALA PG retained some binding, suggesting context dependence to the ability of the LALA PG mutation to eliminate Fc receptor binding (Fig. S1A). Similar results were observed when testing for binding to orthologous murine Fc receptors, specifically murine FcyRIIb, FcyRIII, and FcyRIV (Fig. 2C; Fig. S1B).

**Fig 3:**
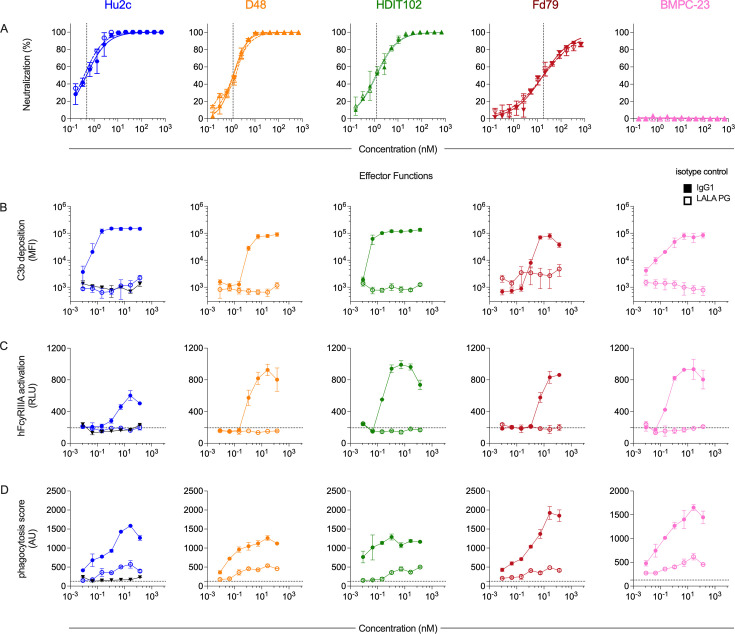
Functional characterization of WT and Fc-engineered gB-specific mAbs. (**A**) Neutralization activity of each gB-specific mAb (columns) against HSV-1 st17 by plaque reduction assay. Dashed vertical line indicates EC_50_ value. (**B–D**) Antibody effector functions. Median fluorescent intensity (MFI) of antibody-dependent deposition of complement cascade factor C3b deposition (**B**), relative light units (RLU) of signaling induced by activation through human FcγRIIIA (**C**), and phagocytic activity (**D**) in arbitrary units (AU) for each gB-specific mAb variant and isotype control (black). Error bars represent standard deviation from the mean. Experiments were performed in technical and 2–3 biological replicates. IgG1 and LALA PG forms are depicted in solid lines with filled symbols and dotted lines with hollow symbols, respectively.

Although the mAbs in the panel had similar antigen recognition profiles, they differed in direct antiviral activity. Hu2c, HDIT102, and D48 all potently neutralized HSV-1 in a plaque reduction neutralization assay (Fig. 3A; Fig. S2). Fd79, on the other hand, neutralized HSV-1 less potently than the other mAbs. Regardless of the mAb dose tested, BMPC-23 did not neutralize HSV (Fig. 3A; Fig. S2). Lastly, we characterized the *in vitro* effector functions of these mAbs. We profiled their ability to induce complement deposition and phagocytosis, as well as FcyRIIIA activation as a surrogate for ADCC activity, for IgG1 and Fc knockout variants (Fig. 3B through D). Each gB-specific IgG1 mAb mediated effector functions, while each LALA PG variant was unable to mediate either complement deposition (Fig. 3B) or FcyRIIIA activation (Fig. 3C). Detectable phagocytic activity was maintained for LALA PG variants, but this activity was greatly diminished compared to IgG1 counterparts (Fig. 3D). Importantly, the LALA PG versions of each gB-specific mAb retained equivalent binding and neutralization potencies as their respective IgG1 counterparts. These results broadly indicate the suitability of the panel of gB-specific mAbs to define mechanisms of *in vivo* antibody-mediated protection from nHSV.

### gB-specific mAbs require effector functions to protect neonatal mice from HSV-1 infection

**Fig 4:**
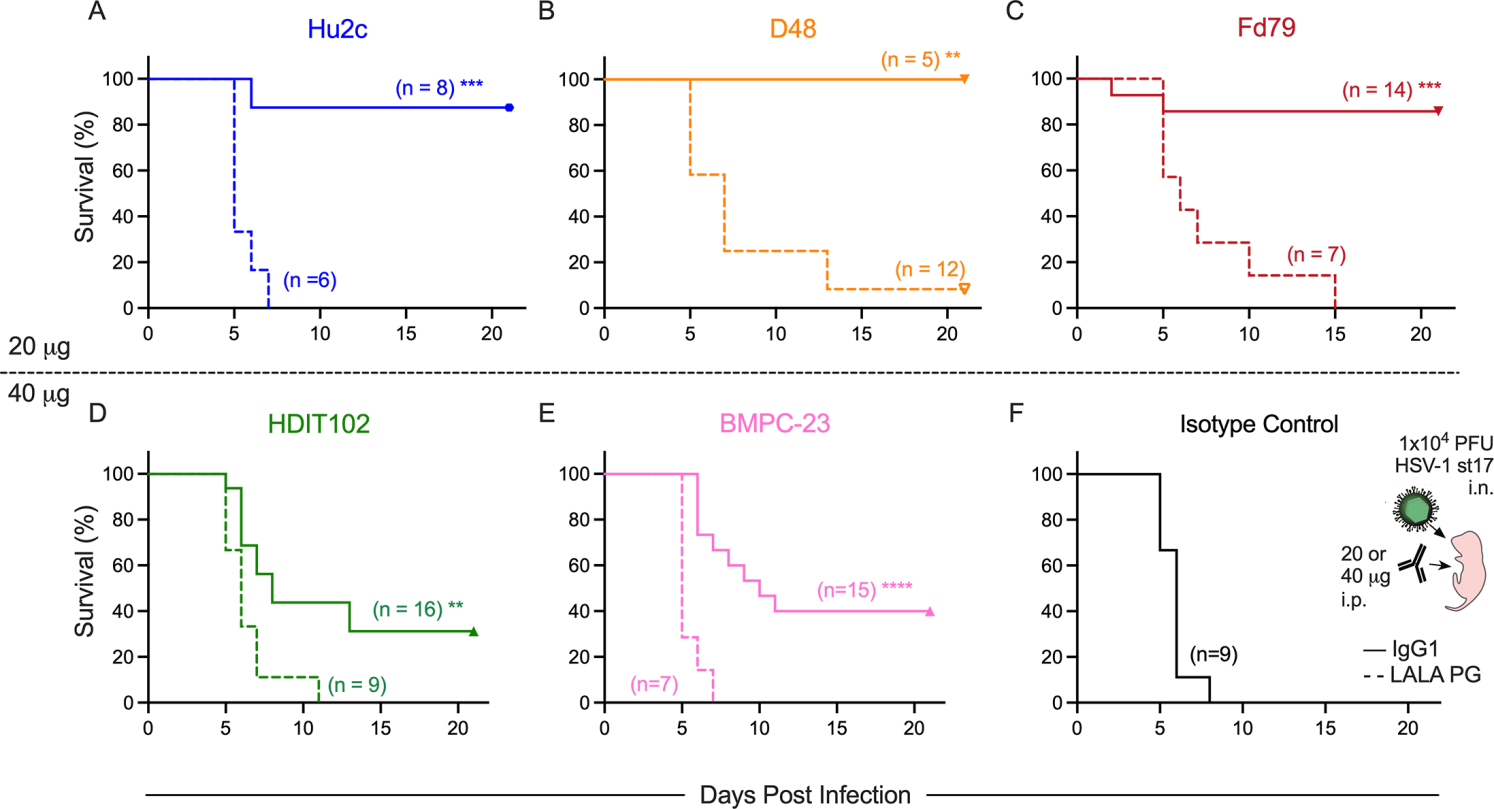
HSV gB-specific mAbs require effector functions to protect neonatal mice. (**A–F**) gB-specific mAbs were delivered intraperitoneally to 2-day-old C57BL/6J mice immediately before a lethal (1 × 10^4^ PFU) challenge with HSV-1 st17. (**A–C**) Survival of pups given 20 µg of WT IgG1 (solid line) or LALA PG KO (dashed line) forms of Hu2c (**A**), D48 (**B**), or Fd79 (**C**). (**D–F**) Survival of pups following 40 µg of WT IgG1 or LALA PG KO mAbs. Pups were given HDIT102 (**D**), BMPC-23 (**E**), or an isotype control mAb (**F**). Number of mice in each condition is reported in the inset. Statistical significance between WT IgG1 and LALA PG KO mAbs is reported in the graph as determined by the log-rank (Mantel-Cox) test. (*P < 0.05, **P < 0.01, ***P < 0.001, and ****P < 0.0001.)

To test protection against nHSV, 2-day-old C57BL/6J pups were injected intraperitoneally (i.p.) with 20 (Fig. 4A through C) or 40 µg (Fig. 4D and E) of the IgG1 versions of each mAb and immediately challenged with 1 × 10^4^ PFU of HSV-1 strain 17 (st17) intranasally (i.n.). At the 20 µg dose, both Hu2c and D48, which robustly neutralize HSV-1, provided nearly complete protection following HSV-1 challenge (Fig. 4A and B). Fd79, which had lower neutralization potency than either D48 or Hu2c, provided equivalent protection (Fig. 4C). Even at twice this dose, the non-neutralizing mAb, BMPC-23, in contrast, only protected about 40% of pups (Fig. 4E). HDIT102, despite exhibiting similar neutralization potency as D48 and binding the same epitope as Hu2c, also protected only 40% of animals at the 40 µg dose (Fig. 4D), suggesting that neither *in vitro* neutralization potency nor epitope specificity is sufficient to fully explain the degree of protection afforded by neutralizing gB-specific mAbs. In contrast, all animals treated with 40 µg of isotype control succumbed to infection (Fig. 4F). These results indicate that while neutralization potency is associated with robust protection, it is insufficient to explain the varying degree of protection observed across the IgG1 mAb panel.

Next, to investigate the extent to which neutralizing mAbs required effector functions for protection, pups were treated with the LALA PG versions of each mAb at the same doses used for their IgG1 versions. While the non-neutralizing mAb BMPC-23 showed an expected dependence on binding to FcyR, all of the neutralizing mAbs, regardless of neutralization potency, required effector functions to be protective in a neonatal mouse model of HSV-1 infection. These results show that viral neutralization alone is insufficient to mediate protection against HSV-1 infection. At the same time, Fc function alone is also insufficient for complete protection, as indicated by the inability of BMPC-23 IgG1 to match the robust protection afforded by D48, Hu2c, and Fd79, even at a higher dose.

### Degree of protection depends on dose and relates to neutralization potency, but effector functions are critical even at high doses of a potently neutralizing mAb

**Fig 5:**
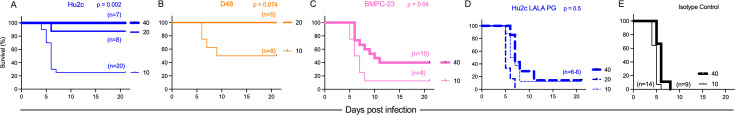
Protection relates to dose and neutralization potency but can require effector function even at high dose. gB-specific mAbs at the indicated dose (µg) were delivered i.p. to 2-day-old C57BL/6J mice prior to a lethal i.n. infection with 1 × 10^4^ PFU of HSV-1. Survival of mice following treatment with Hu2c IgG1 (**A**), D48 IgG1 (**B**), BMPC-23 IgG1 (**C**), Hu2c LALA PG (**D**), or an isotype control mAb (**E**). Number of mice in each condition is reported in inset. Statistical significance between highest and lowest doses tested as determined by the log-rank (Mantel-Cox) test. For ease of comparison across doses, some data are reproduced from Fig. 4.

We next tested additional doses (10–40 µg) for some of the mAbs (Fig. 5) as dose has been shown to influence the mechanisms of antibody-mediated protection (15, 31). For all gB-specific IgG1 mAbs tested at multiple doses, but not the isotype control mAb, the higher dose resulted in greater survival, though testing was not always sufficiently powered to show statistical significance (Fig. 5A through C and E). The degree of protection afforded by mAbs was imperfectly related to their *in vitro* neutralization potency: whereas Hu2c exhibited the most potent activity *in vitro* (Fig. 3; Fig. S2), D48 provided at least as much benefit *in vivo* (Fig. 5A and B). The limited protection afforded by BMPC-23, the non-neutralizing mAb, at the 40 µg dose was reduced further at a 10 µg dose (Fig. 5C).

We previously demonstrated that mAbs specific for gD can exhibit dose-dependent, route-dependent, and virus strain-dependent impacts on mechanisms of protection (15, 32). To begin to explore whether a high mAb dose could overcome the requirement for mAb effector function observed for this panel of gB-specific mAbs, Hu2c was tested in LALA PG form across the full dose series. In contrast to its IgG1 form, Hu2c LALA PG did not demonstrate dose-dependent protection (Fig. 5D), suggesting that increasing the dose of this neutralizing mAb over this range was insufficient to compensate for the loss of binding to FcyR, resulting in an inability of this potently neutralizing mAb to protect against nHSV.

### Robust cross-strain protection afforded by AAV expression of even a poorly neutralizing mAb

**Fig 6:**
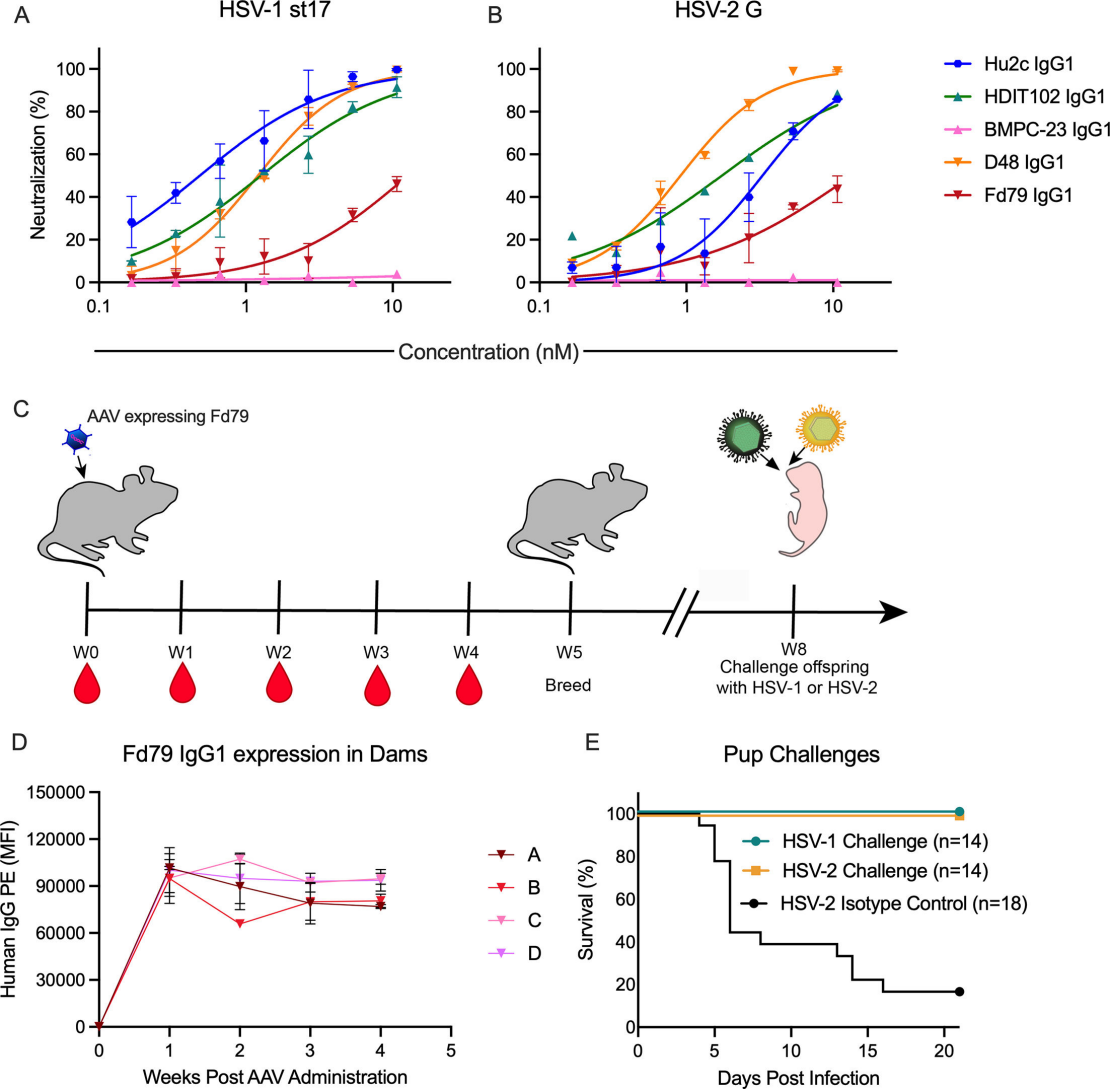
Broad intergenerational protection can be accomplished through vectored delivery of a gB-specific mAb. (**A and B**) Neutralization potencies of gB-specific mAbs against HSV-1 st17 (**A**) and HSV-2 G (**B**). (**C**) Experimental overview and study design in which AAV encoding the gB-specific mAb, Fd79, on a human IgG1 backbone was administered intramuscularly to four female mice prior to breeding and viral challenge of pups. (**D**) Expression of AAV-expressed Fd79 in the sera of female mice at weeks 0 through 4 post-AAV administration. Data present levels of Fd79 in the sera of each of four female mice (**A–D**) over time. (**E**) Survival of offspring of AAV-treated dams challenged with 1 × 10^4^ PFU of HSV-1 st17 or 300 PFU of HSV-2 G on day 2 postpartum.

Having established that direct administration of gB-specific mAbs to pups resulted in dose-dependent protection, we wished to address whether alternative mAb delivery systems could also be efficacious and if protection across HSV serotypes could be observed. Genes to express Fd79 in IgG1 form were administered by vectored immunoprophylaxis (VIP), in which mAb is expressed and secreted from muscle cells *in vivo* following AAV-vectored delivery. With no known epitope, no structure, and limited *in vivo* data (28, 33, 34), the least understood mAb of those explored in this study, Fd79, was selected for delivery via AAV. Moreover, the fact that this relatively poorly neutralizing mAb (Fig. 6A and B) was just as protective against HSV-1 as potent mAbs like D48 and Hu2c warranted further examination of this mAb. Female mice received one intramuscular injection of a nonintegrating AAV8 vector encoding Fd79 (Fig. 6C). *In vivo* expression of Fd79 was confirmed by collecting sera weekly from transduced mice (Fig. 6D). All four dams had robust and stable expression of Fd79 *in vivo* for at least 1 month (Fig. S5). We then assessed whether the expressed mAb was trans-generationally transferred and protective to offspring against HSV challenge, mimicking the protection afforded by maternal HSV seropositivity in humans against neonatal disease.

Offspring of AAV-treated dams were challenged with 1 × 10^4^ PFU of HSV-1 st17 or 300 PFU of HSV-2 stG on day 2 of life. All the progeny of Fd79 AAV-transduced dams were completely protected from HSV-1-mediated and HSV-2-mediated mortality (Fig. 6E). These findings indicate that *in vivo* expression of Fd79 and subsequent transfer to pups is robustly protective and therefore represents an alternative option to single bolus mAb delivery to protect at-risk neonates from HSV infection. Together, these results indicate that diverse gB-specific mAbs protect against nHSV infection and that the degree of protection depends on mAb dose, viral neutralization potency, and antibody effector functions.

## DISCUSSION

Antibodies represent promising candidates for the prevention and treatment of viral infections, especially when efficacious vaccines are unavailable. Determining the dominant mechanisms of Ab-mediated protection is a critical step in the design and optimization of potential mAb therapies. In this study, mAb protective efficacy was dependent on dose, effector functions, and viral neutralization potency. While viral neutralization likely contributes to mAb-mediated protection, the ability for neutralizing gB-specific mAbs to mediate Fc effector functions was unexpectedly crucial. Hu2c, which potently neutralizes HSV-1, required Fc effector functions to be efficacious at all doses tested. Fd79 poorly neutralizes HSV-1 *in vitro* but provided comparable protection to Hu2c *in vivo* and similarly required effector functions in order to be protective. HDIT102, which recognizes an epitope that overlaps with Hu2c and neutralizes HSV-1 with potency intermediate to Hu2c and Fd79, failed to protect most pups following challenge with HSV-1. D48, like Hu2c, also required effector functions to protect neonatal mice from HSV-mediated mortality. These results indicate that neutralization potency, at least as defined by plaque reduction assays *in vitro*, may not be the dominant mechanism of protection even when neutralizing mAb is present at high doses. In contrast, BMPC-23, the non-neutralizing mAb, only mediated partial protection from HSV-1 even at the highest dose tested—indicating that Fc function alone is only partially protective for this gB-specific mAb at the conditions tested.

Our results differ from previous studies using BMPC-23 in adult mice, in which BMPC-23 robustly protected adult mice from HSV-2-mediated mortality (21). Our challenge models, however, differ. Kuraoka et al. used adult vaginal or skin challenge models and administered mAb in advance of challenge, whereas we used intranasal infection in neonatal mice using different HSV serotypes and administered mAb and challenge inoculum simultaneously at a distal site. Furthermore, maximum mAb doses in these studies varied by an order of magnitude. The increased mAb doses evaluated in Kuraoka et al. may account for the incomplete protection observed here. Moreover, the ability for a mAb to mediate effector functions may be impaired in neonates, thus altering the relative importance of antiviral antibody activities. Collectively, these as well as other differences may combine to influence both the degree and means of protection, indicating that challenge context directly influences observed mechanisms of antibody-mediated protection, thus informing conclusions on humoral immunity against HSV across the lifespan.

As we have observed previously (14, 15, 32), Ab dose was a critical determinant for understanding the dominant mechanism(s) of protection against HSV-mediated mortality. We saw an apparent dose-dependent increase in protection against nHSV mortality for the direct administration of mAb to pups. At the lowest dose tested (10 µg/pup), none of the IgG1 mAbs tested were able to fully protect pups from HSV-1-mediated mortality, but protection was improved by increasing the mAb dose. Despite the dose-dependent increase in protection afforded by Hu2c IgG1, the consistent inability of Hu2c LALA PG to provide protection indicated that Hu2c requires effector functions to be protective even when given at a high dose. These findings differ from our previous work with gD-specific mAbs (15), in which viral neutralization was the dominant mechanism of protection at high mAb doses and effector functions were only required at “subneutralizing” mAb doses. It is likely that different glycoprotein targets may be variably susceptible to different mechanisms of Ab-mediated protection, which is critical information for both therapeutic and vaccine design. Together, these results indicate that direct administration of gB-specific mAbs represents a promising approach to protect vulnerable neonates.

While demonstrating their importance, our data do not enable us to pinpoint the relative contributions of different effector functions. The LALA PG mutant abrogates binding to all FcγR and to C1q, imparting a global reduction in effector function. Other experiments, such as with targeted knockouts of different cell types, the complement cascade, or individual receptors, would be needed to parse the contributions of each distinct effector function. Such experiments were not pursued, as evidence from mouse models (35) and humans (36) suggests that the balance of different functions will differ in association with host genetics. Our prior work with HSV gD-specific mAbs also shows that this balance can also differ between virus strains (15, 32). In this context, further refinement of the mechanism is expected to impact the generalizability of refined insights.

Other limitations include only testing mAb efficacy against a single laboratory strain of HSV-1, which may not fully recapitulate virulence and pathogenesis of circulating HSV strains (37). Moreover, we focused on HSV-1 in the direct administration experiments as it has become a major cause of neonatal disease in the United States (38) and has a global seroprevalence of 66% (39), indicating a high burden of disease. Whether our findings translate to HSV-2 will have to be determined. Lastly, while mice are a useful model system in which to perform preclinical experiments, they differ from humans in numerous ways, especially in regard to FcyR distribution and expression on immune cells (40), even in models in which human FcyR are knocked in to native mouse FcyR loci (41). These differences may impact clinical translation and the importance of different Ab-mediated mechanisms of protection in humans.

Beyond direct administration of mAbs, we also demonstrated prolonged delivery of mAb to pups following VIP treatment of dams. Here, we observed that Fd79 is efficiently expressed *in vivo* and that its maternal transfer protects offspring from mortality caused by HSV-1 and HSV-2. In this system, mAb is continuously expressed and transferred across the placenta prior to birth to distribute throughout the pup (14), mimicking the protection afforded by maternal seropositivity in humans (25) and maternal vaccination strategies in mice (42). Moreover, unlike in the direct administration experiments in which mAbs are only delivered once, pups are likely continually supplied with AAV-expressed mAb through nursing, which is a robust mechanism of Ab transfer in rodents (43).

While we observed a clear role for Fc function in mediating protection against nHSV infection, the relatively minor role for viral neutralization was surprising. These data potentially align with failed vaccine trials for HSV in humans. A major vaccine trial that used gD-2 and gB-2 as targets induced robust neutralizing responses, but Abs with little to no Fc functional capacity (44) ultimately failed to meet efficacy criteria and advance beyond clinical trials. Consistent with preclinical studies of protective vaccines that fail to induce neutralizing antibodies (19, 20), our findings in understanding mAb mechanism of action against various HSV antigen targets have demonstrated the importance of Fc effector functions for protection (15, 32). It is possible that vaccine strategies that robustly induce effector function responses like ADCC, in addition to viral neutralization, will be more efficacious against HSV infections. Existing preclinical and clinical data suggest the importance of vaccine strategies for HSV that induce effector functions (7, 45–47). Other considerations include the ability for HSV-specific mAbs to block cell-to-cell spread, a key mechanism of pathogenesis and immune evasion for the virus (48–51). Hu2c, HDIT102, and D48 block cell-to-cell spread *in vitro* but notably require significantly more antibody to block cell spread than entry (26, 27, 52, 53). Variation in this activity may contribute to differences observed between neutralization potency and *in vivo* efficacy, as blocking cell-to-cell spread has been reported to correlate with protection against disease in the guinea pig model (54).

In summary, this study demonstrates that administration of HSV gB-specific mAbs, through either recombinant protein or vectored delivery, potently protects neonatal mice from HSV-mediated mortality. Efficacy was directly influenced by Ab dose, neutralization potency, and Fc effector functions, demonstrating that mAbs with polyfunctional profiles are promising candidates for HSV treatment and prevention, particularly for neonatal infections.

## MATERIALS AND METHODS

### Mouse procedures and viral challenges

C57BL/6J (B6) mice were either purchased from The Jackson Laboratory or bred in-house in accordance with Institutional Animal Care and Use Committee-approved protocols. mAbs were administered via the intraperitoneal route to 2-day-old pups via a 25 µL Hamilton syringe in a 20 µL volume under 1% isoflurane anesthesia. The viral strains used in this study were HSV-1 st17syn+ (55) and HSV-2 G (56) (provided by Dr. David Knipe). Viral stocks were prepared using Vero cells as described elsewhere (57, 58). Newborn pups were infected i.n. on day 2 of life with 1 × 10^4^ PFU of HSV-1 st17 or 300 PFU of HSV-2 stG in a 5 µL volume under 1% isoflurane anesthesia. Pups were then monitored and weighed daily through day 21 post-infection. Endpoint criteria for the viral challenge experiments were defined as excessive morbidity (hunching, spasms, and/or paralysis) and/or >10% weight loss from the previous measurement.

### Monoclonal antibodies

Hu2c (26, 27), HDIT102 (27), Fd79 (28), BMPC-23 (21), and D48 (29) VH and VL sequences were collected from published sequences or published structures and synthesized by Twist Biosciences as full-length IgG1 heavy chains and Kappa (Hu2c, Fd79, BMPC-23, and D48) or Lambda (HDIT102) constant light chains. Recombinant DNA was cloned into pCMV expression vectors. Fc variants of each gB-specific mAb were generated via overlap extension PCR, combining the VH for each mAb with constant domains containing the specific Fc mutations. All expression plasmids containing mAb heavy or light chain sequences were sequence confirmed via Sanger or whole plasmid sequencing (Azenta/Genewiz). mAbs were expressed via the co-transfection of heavy and light chain sequences in ExpiCHO cells (ThermoFisher) in accordance with the manufacturer’s protocols. At 8–10 days post-transfection, cultures were spun for 3 h at 3,000 RCF to pellet the cells. Supernatants were then sterile filtered (0.22 µm). All mAbs were purified using custom packed protein A columns (Cytiva) or protein A/G plus agarose (ThermoFisher) and eluted with 100 mM glycine (pH 3.0). Eluates were immediately neutralized using 1 M Tris-HCl (pH 8.0). mAbs were concentrated and buffer-exchanged into PBS using Amicon 30 kDa cut-off filters. mAbs were passed over endotoxin removal columns, aliquoted, and snap-frozen before being stored at −80°C until use. ChimeraX (59) was used to make representative figures with the known epitopes of the gB-specific mAbs used in this study using the determined structures of HSV-1 prefusion (60) and postfusion (61) gB.

### Measurement of binding to HSV-1 gB

Recombinant HSV-1 gB antigen (provided by Dr. Gary Cohen) was coupled to MagPlex (Luminex) beads as previously described (62). gB-specific mAbs were serially diluted in 1× PBS with 0.1% bovine serum albumin (BSA) and 0.05% Tween-20 and incubated with antigen-coupled beads overnight at 4°C with constant shaking. Antibody immune complexes were washed once before being incubated with an anti-human IgG-PE secondary antibody (Southern Biotech) for 1 h at room temperature with constant shaking. Beads were washed a second time and then analyzed on the xMap system (Luminex). The median fluorescence intensity of at least 10 beads/region was recorded. An isotype control antibody and buffer-only well control were used to determine assay background.

### Measurement of antibody binding to human and murine Fc receptors

Serially diluted gB-mAbs were incubated with gB and anti-human IgG MagPlex beads as described above. Beads were washed before being incubated with recombinant biotinylated human Fc gamma receptors (63) (Duke Human Vaccine Institute) or murine Fc gamma receptors (Acro Biosystems) that were tetramerized with streptavidin-PE for 1 h. The beads were washed and analyzed on the xMap system. The median fluorescence intensity of at least 10 beads/region was recorded. An isotype control antibody and a buffer-only control were used to determine antigen-specific binding and assay background signal. Area under the curve was calculated using Prism 10 (GraphPad).

### Viral neutralization

Viral neutralization of HSV-1 and HSV-2 was performed via plaque reduction neutralization as previously described (15, 32). Briefly, serially diluted mAbs were incubated with 100 PFU of HSV-1 st17 or HSV-2 stG for 1 h at 37°C before being added to confluent Vero cell monolayers grown in six-well plates (Corning). Virus:mAb immune complexes were incubated with cells for 1 h at 37°C with shaking every 15 min. After the incubation, methylcellulose overlay was added to the cells. Plates were incubated for 48 h (HSV-1) or 72 h (HSV-2) at 37°C with 5% CO_2_. After incubation, the overlay was removed, and cells were fixed with 1:1 methanol:ethanol for 30 min at RT. The plates were then stained with 12% Giemsa overnight. The stain was removed, and plaques were counted on a light box. Viral neutralization (%) was calculated as ([number of plaques in virus-only control well − number of plaques counted at mAb dilution]/number of plaques in virus-only control well) × 100. Assays were performed in technical and 2–3 biological replicates.

### Antibody-dependent complement deposition

Antigen beads were prepared by coupling HSV-1 gB to coded Magplex superparamagnetic carboxylated magnetic microparticles (Luminex Corp) using carbodiimide cross-linking chemistry as previously described (63). Antigen beads were diluted in assay buffer to 500 beads/well and then incubated with serially diluted antibody for 2 h at room temperature with shaking. Following antibody binding, the plate was washed with assay buffer (PBS with 0.01% BSA and 0.005% Tween-20) and treated with 1:100 dilution of guinea pig serum (Cedarlane). The plate was sonicated and vortexed to allow for mixing of beads and antibodies before being sealed and incubated at 37°C for 30 min. Following serum incubation, the plate was washed on a plate washer with assay buffer. For C3b detection, 40 μL of 0.65 μg/mL of biotin-conjugated anti-guinea pig C3 conjugated with PE (ICL, GC3-60B-Z) was added to the plate and incubated for 1 h at room temperature with shaking. The plate was then washed using an automatic plate washer, and beads were resuspended in 50 μL of sheath fluid. Data were acquired on a Luminex FLEXMAP 3D Instrument System, which detects beads and measures PE fluorescence to calculate the median fluorescent intensity level of each analyte.

Heat-inactivated guinea pig serum (heated for 30 min at 56°C) was used as a negative control. An HIV-specific IgG1 (VRC01) was used as an isotype control.

### Antibody-dependent cellular phagocytosis

Antibody-dependent cellular phagocytosis was performed as previously described (64). Briefly, fluorescent antigen beads were prepared by conjugating HSV-2 gB to yellow-green carboxylate beads (ThermoFisher). gB mAbs were serially diluted fourfold in culture medium and incubated with fluorescent antigen beads for 2 h at 37°C to allow for immune complex formation. After incubation, THP-1 cells (25,000/well) were added to the immune complexes, and the plate was incubated for 4 h at 37°C. The plate was then washed with cold PBS and fixed with 4% paraformaldehyde. Cells were run on a NovoCyte Advanteon flow cytometer (Agilent). Phagocytosis score was calculated as the (percentage of FITC+ cells) × (geometric mean fluorescence intensity of the FITC+ cells)/10,000. Buffer-only wells served as a negative control. The assay was performed in technical replicate with two biological replicates.

### Antibody-dependent cellular cytotoxicity (ADCC)

Antigen-coated plate was prepared by incubating the plate with 1 μg/mL of HSV-2 gB and storing it overnight at 4°C. The plate was then washed and incubated with 200 μL/well of blocking buffer for 1 h at room temperature. Antibodies were serially diluted. Jurkat-Lucia cells (Invivogen) were spun down and resuspended in assay medium at 1,000,000 cells/mL. Following the 1 h incubation, the blocked plate was washed and incubated with 100 μL of antibody dilution for 30 min. Cells were added to the wells (100,000/well), and the plate was incubated overnight at 37°C with 5% CO_2_. Following a 24 h incubation, 25 μL of supernatant from each well was transferred to an opaque white 96-well plate, and 75 μL of Quanti-Luc reagent (Invivogen) was added to each well. Luminescence was immediately read on a SpectraMax Paradigm plate reader (Molecular Devices) using a 1 s integration time. Kinetic readings at 0 min, 2.5 min, and 5 min were measured, and the mean reading was noted. Cell Simulation Cocktail (eBioscience) was used as a positive control. VRC01 was used as a negative control. The assay was performed with two biological replicates.

### AAV production and mouse procedures

AAVs encoding the heavy and light chain sequences of Fd79 on a human IgG1 backbone were constructed as previously described. A single 40 µL injection of 1 × 10^11^ genome copies of the AAV was administered into the gastrocnemius muscle of female B6 mice as previously described (14). Dams were bled weekly via the mandibular vein with a 5 mm lancet. Blood was allowed to clot by stasis at room temperature for 30 min and then spun at 2,000 RCF for 20 min at 4°C. Sera were collected and stored at −20°C. AAV-driven antibody expression was verified using the magnetic bead-based assay as described above. Serially diluted sera were incubated with gB and anti-human IgG capture beads and detected with an anti-human IgG-PE secondary antibody (Southern Biotech) and analyzed via the xMap system (Luminex).

## Data Availability

Data are available on reasonable request to the corresponding authors.

## SUPPLEMENTAL MATERIAL

Supplemental figures jvi.00050-26-s0001.pdf Figures S1 to S5.

ASM does not own the copyrights to Supplemental Material that may be linked to, or accessed through, an article. The authors have granted ASM a non-exclusive, world-wide license to publish the Supplemental Material files. Please contact the corresponding author directly for reuse.
