# Supplementary material for: Antibodies targeting HSV glycoprotein B require effector functions to protect neonatal mice
Source: J Virol. 2026 Mar 9;100(4):e00050-26. doi: 10.1128/jvi.00050-26 (PMC13098214; doi:10.1128/jvi.00050-26)
Supplement: Supplemental figures — Figures S1 to S5. [file jvi.00050-26-s0001.pdf]

## Supplemental Figures and Legends

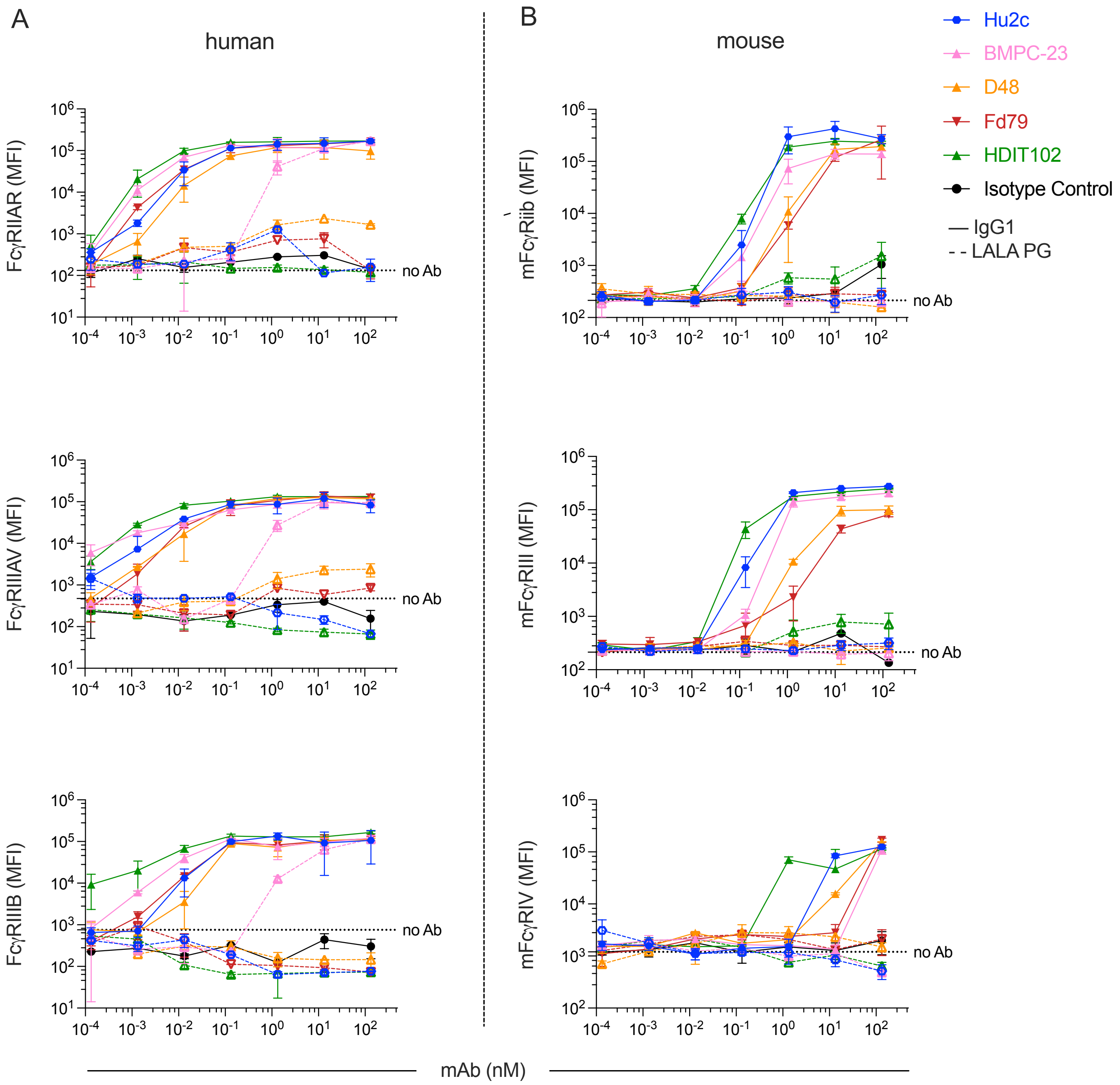


**Supplemental Figure 1 (Related to Figure 2): FcyR binding of gB-specific mAbs and their Fc-engineered variants. A-B**. Median fluorescent intensity (MFI) of tetramerized human (**A**) and mouse (**B**) FcγR bound to gB-conjugated microspheres following incubation with titrations of IgG1 (solid) and LALA PG (dashed) forms of each indicated mAb (color). Error bars represent standard deviation from the mean. Experiments were performed in technical and 2-3 biological replicates. Dotted horizontal line indicates signal observed in the absence of mAb.

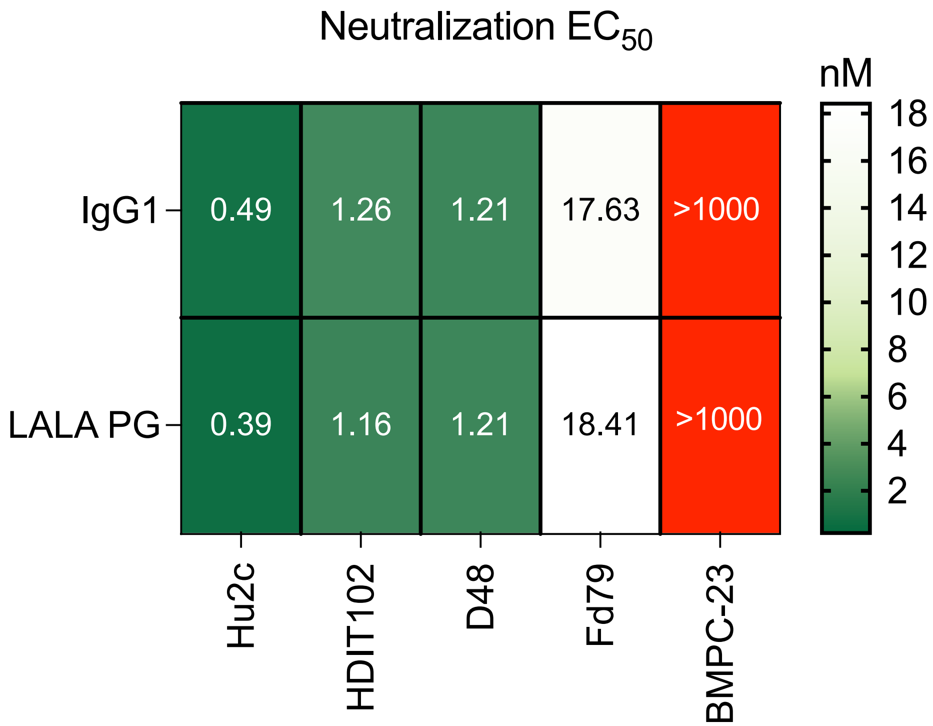


**Supplemental Figure 2 (Related to Figure 3): Neutralization potency.** Heatmap of neutralization midpoint Effective Concentration ($EC_{50}$) values for HSV-1 between the IgG1 and LALA PG Fc forms of each indicated gB-specific mAb.

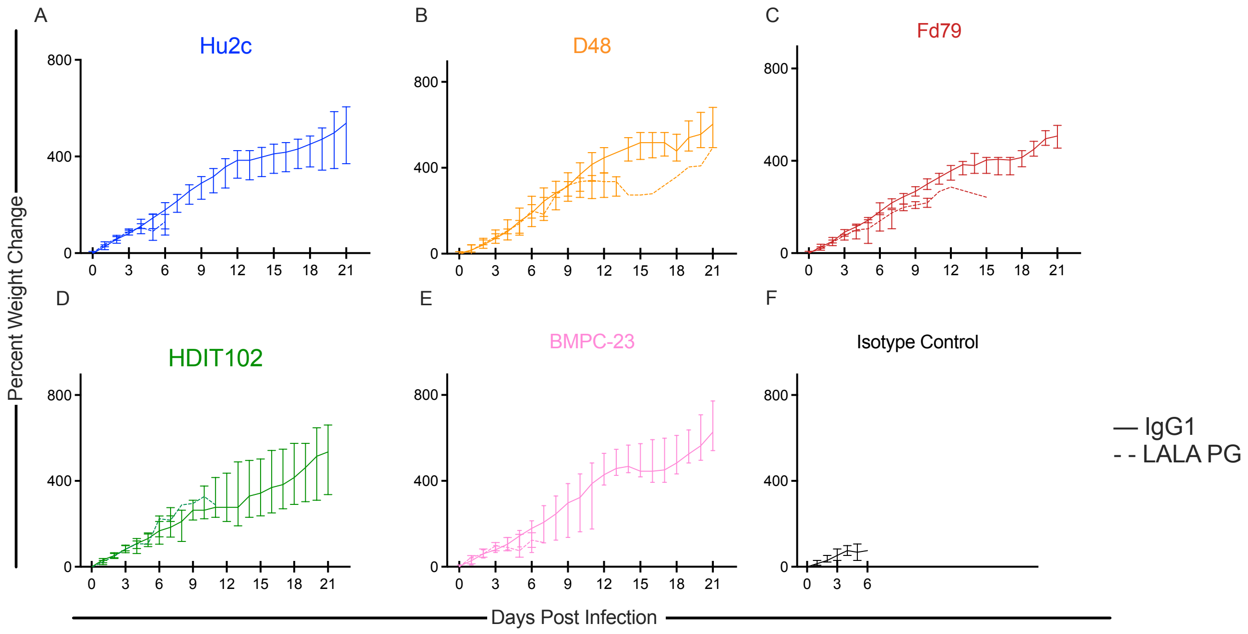


**Supplemental Figure 3 (Related to Figure 4) Percent weight gain of mice following HSV-1 challenge.** Mice were weighed daily for 21 days post challenge and treatment with indicated mAb at either 20 (top row) or 40 (bottom row) μg dose. Error bars represent the standard error of the mean.

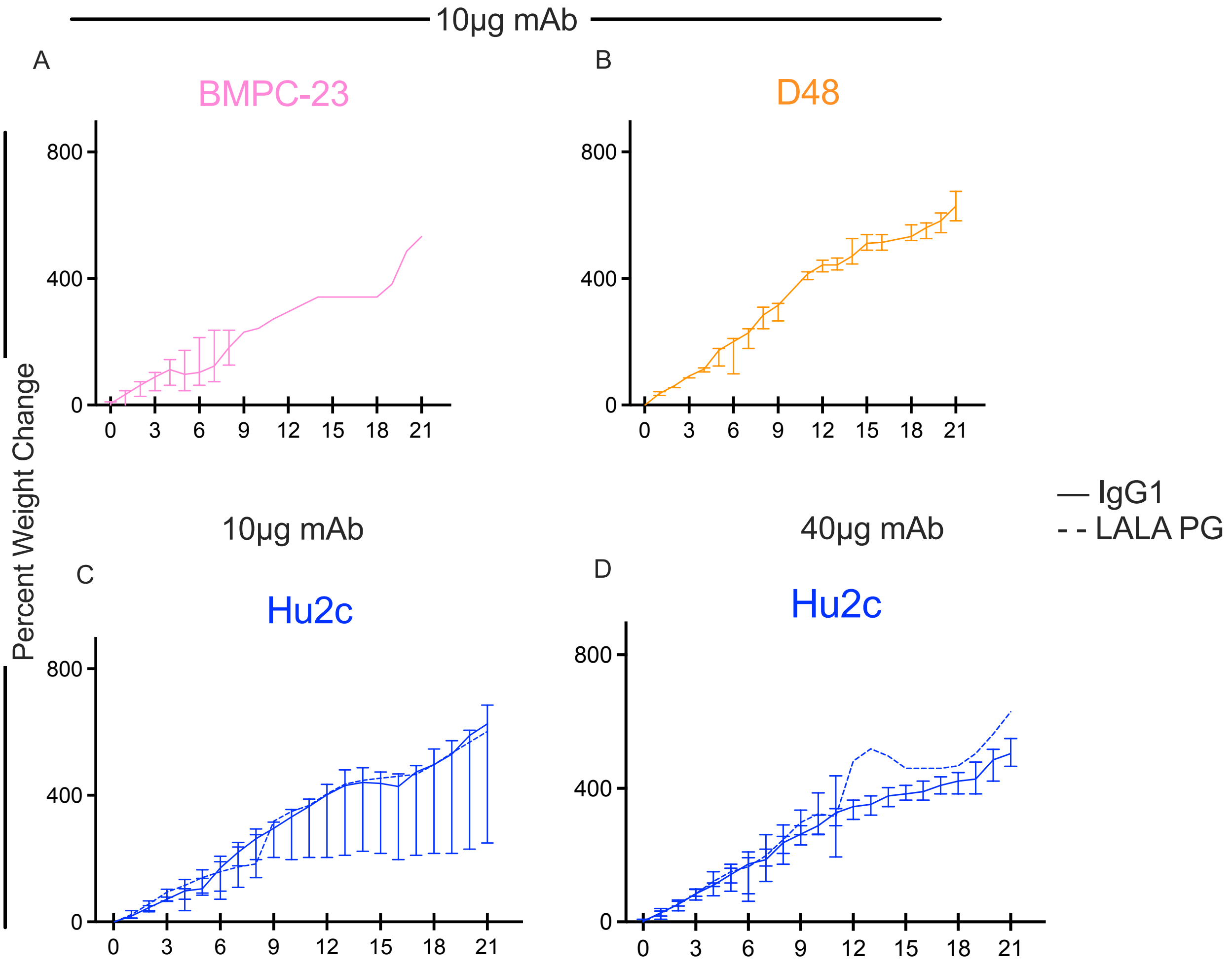


**Supplemental Figure 4 (related to Figure 5)**. **Percent weight gain of mice given alternative doses of gB-specific mAbs following HSV-1 challenge.** 2-day-old C57BL/6J mice received 10 (**A-C**) or 40 µg (**D**) of the indicated gB-specific mAbs or isotype control delivered i.p. immediately before a lethal challenge with $1x10^4$ PFU of HSV-1 st17. Mice were monitored for 21 days post infection and weighed daily. Error bars represent the standard error of the mean.

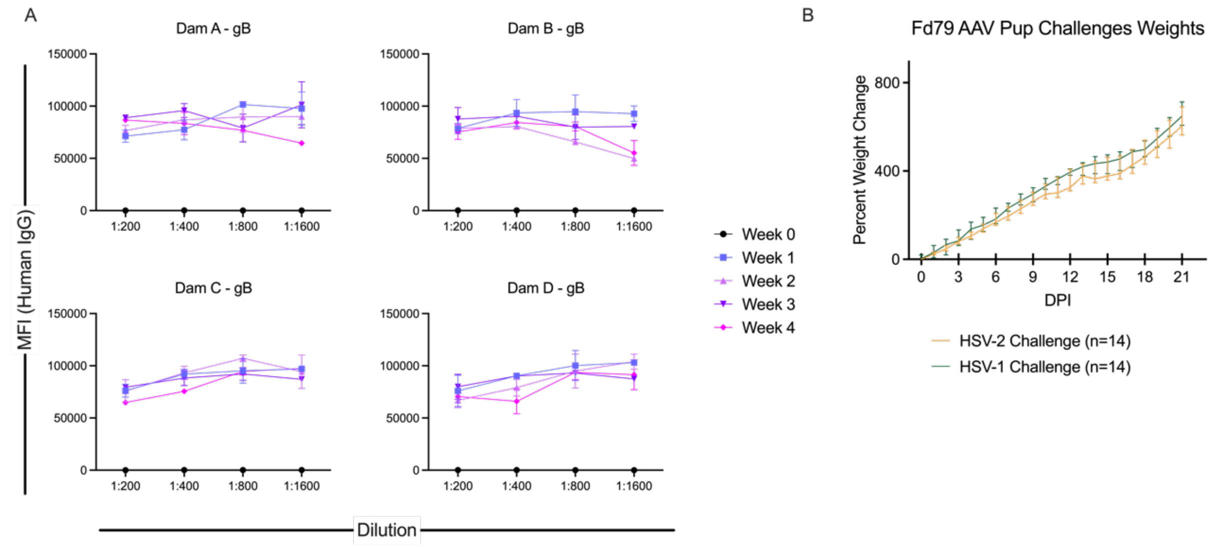


**Supplemental Figure 5 (related to Figure 6) AAV-expression of Fd79 is stable over time and protects offspring from HSV-mediated morbidity and mortality. A**. *In vivo*-expressed Fd79 was detected in the sera of 4 female mice at weeks 0-4 post transduction. Error bars represent standard deviation from the mean **B**. Percent weight gain of offspring of Fd79-AAV-transduced dams following HSV-1 or HSV-2 challenge. Error bars represent standard error of the mean.
